# Dynamics in perioperative neutrophil-to-lymphocyte*platelet ratio as a predictor of early acute kidney injury following cardiovascular surgery

**DOI:** 10.1080/0886022X.2021.1937220

**Published:** 2021-06-30

**Authors:** Yang Li, Zhouping Zou, Yunlu Zhang, Bowen Zhu, Yichun Ning, Bo Shen, Chunsheng Wang, Zhe Luo, Jiarui Xu, Xiaoqiang Ding

**Affiliations:** a Department of Nephrology, Zhongshan Hospital, Fudan University, Shanghai, China; b Shanghai Medical Center of Kidney, Shanghai, China; c Shanghai Key Laboratory of Kidney and Blood Purification, Shanghai, China; d Department of Cardiovascular Surgery, Zhongshan Hospital, Fudan University, Shanghai, China; e Department of Critical Care Medicine, Zhongshan Hospital, Fudan University, Shanghai, China

**Keywords:** Acute kidney injury, neutrophils, lymphocyte, platelet, predictor, cardiovascular surgery

## Abstract

**Background:**

In this study, we applied a composite index of neutrophil-lymphocyte * platelet ratio (NLPR), and explore the significance of the dynamics of perioperative NLPR in predicting cardiac surgery-associated acute kidney injury (CSA-AKI).

**Methods:**

During July 1^st^ and December 31^th^ 2019, participants were prospectively derived from the ‘Zhongshan Cardiovascular Surgery Cohort’. NLPR was determined using neutrophil counts, lymphocyte and platelet count at the two-time points. Dose-response relationship analyses were applied to delineate the non-linear odds ratio (OR) of CSA-AKI in different NLPR levels. Then NLPRs were integrated into the generalized estimating equation (GEE) to predict the risk of AKI.

**Results:**

Of 2449 patients receiving cardiovascular surgery, 838 (34.2%) cases developed CSA-AKI with stage 1 (*n* = 658, 26.9%), stage 2–3 (*n* = 180, 7.3%). Compared with non-AKI patients, both preoperative and postoperative NLPR were higher in AKI patients (1.1[0.8, 1.8] vs. 0.9[0.7,1.4], *p* < 0.001; 12.4[7.5, 20.0] vs. 10.1[6.4,16.7], *p* < 0.001). Such an effect was a ‘J’-shaped relationship: CSA-AKI’s risk was relatively flat until 1.0 of preoperative NLPR and increased rapidly afterward, with an odds ratio of 1.13 (1.06–1.19) per 1 unit. Similarly, patients whose postoperative NLPR value >11.0 were more likely to develop AKI with an OR of 1.02. Integrating the dynamic NLPRs into the GEE model, we found that the AUC was 0.806(95% CI 0.793–0.819), which was significantly higher than the AUC without NLPR (0.799, *p* < 0.001).

**Conclusion:**

Dynamics of perioperative NPLR is a promising marker for predicting acute kidney injury. It will facilitate AKI risk management and allow clinicians to intervene early so as to reverse renal damage.

## Introduction

Acute kidney injury (AKI) is a complicated and multifactorial clinical syndrome, which is characterized by a sudden decrease in renal function. Cardiac surgery is the second commonest reason for AKI behind sepsis [1]. It was reported that the incidence of cardiac surgery-associated AKI (CSA-AKI) varied from 5% to 42% [2]. CSA-AKI not only prolongs the length of stay in the intensive care unit (ICU) and hospitalization but increases the cost of care and mortality [3,4]. Early identification of patients at high risk of CSA-AKI and prompt modification of reversible factors will optimize the clinical outcome and avoid unnecessary kidney damage.

Preoperative inflammation is commonly encountered in patients receiving cardiac surgery. It can be caused either by heart infection (such as infectious endocarditis) or other chronic infectious diseases (COPD). In clinical practice, the neutrophil to lymphocyte ratio (NLR) is an easily effective and low-cost biomarker of inflammatory status [5,6]. Previous studies have demonstrated its value in early prediction of infectious conditions [5,7], cancers [8–13] and cardiovascular diseases [14]. As the initiator of inflammation, neutrophils can quickly migrate from peripheral blood circulation to kidney under the action of chemokines. It can result in CSA-AKI by blocking renal micro-vessels and secreting oxygen free radicals and proteases. Besides, platelet-neutrophil aggregates are formed during the inflammatory process [15] and associated with diverse pathologies [16,17]. It also can cause renal damage through vascular lesions and tissue destruction [18]. To this end, NLPR may be the more promising biomarker of inflammation than NLR in CSA-AKI prediction. Although several studies have shown that high NLPR was associated with postoperative AKI in cardiovascular surgery patients [19], there is still no research that considers the dynamics of NLPR in the prediction model. The effect of NLPR on AKI is not a simple linear relationship. It is crucial to delineate the dose-response relationship between NLPR and AKI risk and the cutoff of NLPR for CSA-AKI prediction. This study aims to explore the significance of the dynamic changes in perioperative NLPR for predicting CSA-AKI.

## Methods

### Study design and patient selection

This study was designed as a prospective cohort study based on the ‘Zhongshan Cardiovascular Surgery Cohort’. This cohort was established in 2009 and contained nearly 30,000 subjects with complete demographic and clinical records. We recruited patients who underwent cardiac surgeries in 1 July 2019 to 31 December 2019. The inclusion criteria of participants were as follows: (1) aged over 18 years, (2) underwent cardiac surgery, (3) did not receive renal replacement therapy (RRT) before surgery, and (4) had completed renal and cardiac data. We further excluded those who were under 18 years old, received the heart transplant and RRT, or took less than one serum creatinine (SCr) test. This study was approved by the Institutional Committee of Zhongshan Hospital (B2017-039). Participants were given informed consent prior to recruiting, and their identity information was replaced as a unique code during analysis.

### Data collection

Patients’ demographics were collected through a structured questionnaire. Clinical data with timestamps were extracted from the electronic medical records (EMR). We selected thirty-seven factors for analysis. They were divided into five groups in chronological order: (1) demographics and comorbidity: age, gender, body mass index (BMI), hypertension, diabetes, unstable angina pectoris (UAP), and myocardial infarction (MI) within the last 3 months; (2) preoperative cardio-renal function [within 24 h of admission to hospital]: cardiac surgery history, coronary angiography, New York heart association (NYHA) grade, left ventricular eject fractions (LVEF), pulmonary artery pressure (PAH), SCr, blood urea nitrogen (BUN), estimated glomerular filtration rate (eGFR), serum uric acid (SUA) and proteinuria; (3) perioperative blood routine test [within 24 h of admission]: glucose, albumin, hemoglobin, peripheral blood cell counts including erythrocyte, total leukocyte, neutrophil, lymphocyte and platelet; (4) intraoperative factors: cardiopulmonary bypass (CPB), surgery type, aortic cross-clamp time (ACCT), ultrafiltration volume and blood transfusion; (5) postoperative factors (within 1 h of arrival in the intensive care unit [ICU]): APACHE II score, Euro score and blood routine test. Moreover, we also retrieved the preoperative medication data for patients with higher NLPR to evaluate the effect of antibiotic use on AKI occurrence.

### Definition and classification

According to the 2012 Kidney Disease: Improving Global Outcomes (KDIGO) criteria [20], AKI was defined as the absolute value of the SCr increase ≥0.3 mg/dL (≥26.5 μmol/L) within 48 h or an increase ≥1.5 times baseline levels within 7 days, or a urine output <0.5 mL/kg/h lasting over 6 h. The SCr value on admission was regarded as the baseline level. CSA-AKI was diagnosed by comparing it with the postoperative SCr value. For those receiving multiple SCr tests after surgery, we defined the highest one as the peak value. The equation of NLPR was factorized as:

$$\mathrm{Neutrophil}-\mathrm{to}-\text{Platelet*Lymphocyte Ratio }(\mathrm{NLPR})=\frac{\text{Neutrophil count }(10^{9}/L)*100}{\text{Lymphocyte count }(10^{9}/L)\text{*Platelet count}(10^{9}/L)}$$

It was calculated at two-time points: on admission and within 1 h at ICU. Cardiac surgery was typed into the valve, coronary artery bypass grafting (CABG), aorta, valve + CABG, valve + large vessels and others.

### Statistical analysis

Statistics were conducted by using R 3.6.1 software (R core team). Continuous variables are presented as mean ± standard deviation or median with inter-quartile range (IQR). Categorical variables were expressed in numbers and percentages (‘Hmisc’ package). Missing data were found in less than 10% of the records, mainly focusing on NYHA grade, BMI, and albumin. We imputed the missing value by the methods of multiple imputations (‘mice’ package in R, method=‘rf’, *m* = 5, maxit = 5). Student’s t test and Wilcoxon test were applied to compare the distributional difference of continuous variables. Pearson test and Fisher’s exact test were applied to compare the proportional difference of categorical variables (‘gmodels’ package). Given the prior hypothesis of the dose-response relationship, we used restricted cubic splines with three knots at the 10th, 50th and 90th centiles to flexibly model the association of neutrophil count, lymphocyte count, platelet count, and NLPR with CSA-AKI incidence (‘tidyverse’, ‘rms’ and ‘ggplot2’ packages). Non-linearity was tested by using a likelihood ratio test comparing the model with only a linear term against the model with linear and cubic spline terms. Bounded with the median value, we used a logistic model to calculate odds ratios (ORs) for each 1/10 unit increase in predict variables. Moreover, the generalized estimating equation (GEE) was applied for multivariate analysis, which combined the time-dependent indicators of leukocyte counts and NLPR (‘geepack’ package). We applied the purposeful selection of covariates [21]. In model 1, we only included NLPR in two-time points for univariate analysis. Then we included other factors gradually: (i) model 2: model 1 plus demographic factors; (ii) model 3: model 2 plus preoperative factors; (iii) model 4: model 3 plus intra-/postoperative factors. The predictive power was quantified through the area under the receiver operating characteristic (AUROC) curves (‘pROC’ and ‘ggplot2’ packages). Delong test was used to compare the significance of ROC curves. All statistical tests were two-tailed, and we regarded *p* < 0.05 as the criterion for statistical significance.

## Results

### Baseline characteristics and CSA-AKI incidence

Of the 2618 enrolled participants who underwent cardiac surgeries, 2449 patients met the inclusion criteria and were comprised of formal analysis (Supplement Figure 1). The average age was 57.2 ± 12.8 years old, and male patients accounted for 59.2% (*n* = 1449). According to KDIGO classification, 838 (34.2%) patients developed AKI after surgery. Of them, 658 patients located in Stage-1, and another 131 and 49 cases were Stage-2 and Stage-3, respectively. The rate of AKI-RRT was 1.2% (*n* = 30). Table 1 listed the major factors of CSA-AKI. Patients who were elder age, male, had higher BMI, or suffered from hypertension and diabetes were more likely to occur CSA-AKI. Cardio-surgery history, coronary arteriography, attenuated LVEF were positively associated with CSA-AKI. Given that patients already had preexisting renal dysfunction at admission, the risk of CSA-AKI was also increased. In terms of intraoperative factors, patients who underwent complicated cardiac surgery (aorta, valve combined with CABG, or larger vessels) were more susceptible to CSA-AKI. The application of CPB and blood transfusion, prolonged ACCT, and high ultrafiltration volume also had notable impacts on CSA-AKI. With the increase of APACHE II/Euro score, the postoperative risk CSA-AKI also kept growing.

**Table 1.** Demographic and perioperative factors of CSA-AKI (*n* = 2449).

Variables	AKI	Non-AKI	Stat. value	*p*-Value
Age (year)	59.5 ± 11.0	56.0 ± 13.5	−6.567	<0.001^†^
Gender (male %)	576 (68.7)	873 (54.2)	48. 272	<0.001^#^
BMI (kg/m²)	24.4 ± 3.8	23.4 ± 3.4	−6.386	<0.001^†^
Hypertension (%)	410 (48.9)	550 (34.1)	50.565	<0.001^#^
Diabetes (%)	107 (12.8)	161 (10.0)	4.355	0.037^#^
Unstable angina pectoris (%)	37 (4.4)	74 (4.6)	N/A	0.919*
Myocardial infarction (%)	12 (1.4)	13 (0.8)	N/A	0.202*
Cardiac surgery history (%)	52 (6.2)	56 (3.5)	N/A	0.002*
NYHA grad*e* ≥ 3 (%)	666 (78.5)	1002 (62.2)	75.761	<0.001^#^
Coronary Arteriography (yes %)	551 (65.8)	942 (58.5)	12.272	<0.001^#^
LEVF (%)	60.1 ± 8.9	62.3 ± 7.9	6.320	<0.001^†^
PAH (mmHg)	41.6 ± 13.5	41.1 ± 14.7	−0.751	0.453^†^
BUN (mmol/L)	7.3 ± 5.8	6.3 ± 3.1	−5.566	<0.001^†^
SCr (µmol/L)	90.6 ± 48.8	82.7 ± 61.8	−3.190	0.001^†^
eGFR (ml/min/1.73m²)	80.3 ± 23.3	86.4 ± 19.4	6.908	<0.001^†^
SUA (µmol/L)	394.2 ± 121.1	354.1 ± 105.7	−8.477	<0.001^†^
Proteinuria (yes %)	80 (9.5)	79 (4.9)	19.572	<0.001^#^
Random blood sugar (mmol/L)	5.0[4.6, 5.7]	4.9[4.6, 5.5]	N/A	0.002^‡^
Albumin (g/L)	40.2 ± 4.5	40.6 ± 4.3	2.516	0.012^†^
Surgery type			84.300	<0.001^#^
Valve	463 (55.3)	793 (49.2)		
CABG	73 (8.7)	259 (16.1)		
Aorta	124 (14.8)	146 (9.1)		
Valve + CABG	42 (5.0)	32 (2.0)		
Valve + large vessels	40 (4.8)	59 (3.7)		
Others	96 (11.5)	322 (20.0)		
CPB (yes %)	740 (88.3)	1029 (63.9)	164.064	<0.001^#^
ACCT (min)	68.8 ± 39.7	64.0 ± 55.2	−2.021	0.043^†^
Ultrafiltration volume (L)	2.5[2.0, 3.3]	2.0[1.5, 2.7]	N/A	<0.001^‡^
Blood transfusion (yes, %)	222 (26.5)	127 (7.9)	156.207	<0.001^#^
Plasma (ml)	400[400, 600]	400[200, 400]	N/A	<0.001^‡^
Euro score	3.9 ± 2.4	3.5 ± 2.2	−4.338	<0.001^†^
APACHE II score	8.2 ± 4.2	6.5 ± 3.4	−10.914	<0.001^†^

^†^Refers to Student’s t test (for continuous variables which were normally distributed).

^#^Refers to Pearson test (for binary and unordered categorical variables).

*Refers to Fisher’s exact test (for categorical variables when sample sizes are small).

^‡^Refers to Wilcoxon test (for continuous variables which were non-normally distributed).

ACCT: aortic cross-clamp time; AKI: acute kidney injury; aOR: adjusted odds ratio; CABG: coronary artery bypass grafting; CI: confidence interval; CPB: cardiac pulmonary bypass; CSA-AKI: cardiac surgery-associated acute kidney injury; eGFR: estimated glomerular filtration rate; LVEF: left ventricular ejection fractions; NYHA: New York heart association; PAH: pulmonary arterial hypertension; SCr: serum creatinine; SUA: serum uric acid.

### Dynamics of peripheral blood cell counts and NLPR

The blood analysis was summarized in Table 2. Compared with non-AKI patients, preoperative neutrophil counts (3.9 ± 2.4 vs. 3.5 ± 1.6, *p* < 0.001; aOR 1.07 (95% CI 1.02–1.12), *p* < 0.001), and NLPR (1.1[0.8, 1.8] vs. 0.9[0.7,1.4], *p* < 0.001; aOR 1.15 (95% CI 1.09–1.22), *p* < 0.001) were higher in AKI patients. Lymphocyte (1.7 ± 0.6 vs. 1.9 ± 0.7, *p* < 0.001; aOR 0.74 (95% CI 0.64–0.85), *p* < 0.001) and platelet (182.2 ± 59.6 vs. 196.4 ± 60.9, *p* < 0.001; aOR 0.99 (95% CI 0.99–1.00), *p* < 0.001) counts were negatively associated with AKI risk. Due to the posttraumatic stress response after surgery, blood cell counts changed a lot. While patients with a higher postoperative NLPR still had an increased risk of developing AKI (12.4[7.5, 20.0] vs. 10.6[6.4,16.7], *p* < 0.001; aOR 1.02 (95% CI 1.02–1.03), *p* < 0.001).

**Table 2.** Distribution of peripheral blood cell counts and NLPR in AKI and non-AKI patients (*n* = 2449).

Blood cell counts	AKI	Non-AKI	Stat. value	*p*-Value	aOR (95% CI)^c^	*p*-Value
Pre-surgery (within 24 h of admission)						
Leukocyte (10^9^/L)	6.3 ± 2.5	6.1 ± 1.9	−2.588	0.009^†^	1.03 (0.99–1.08)	0.112
Neutrophil (10^9^/L)	3.9 ± 2.4	3.5 ± 1.6	−4.018	<0.001^†^	1.07 (1.02–1.12)	<0.001
Lymphocyte (10^9^/L)	1.7 ± 0.6	1.9 ± 0.7	4.926	<0.001^†^	0.74 (0.64–0.85)	<0.001
Platelet (10^9^/L)	182.2 ± 59.6	196.4 ± 60.9	5.542	<0.001^†^	0.99 (0.99–1.00)	<0.001
NLPR	1.1[0.8, 1.8]	0.9[0.7, 1.4]	N/A	<0.001^‡^	1.15 (1.09–1.22)	<0.001
Post-surgery (within 1 h of arrival in ICU)						
Leukocyte (10^9^/L)	12.7 ± 3.8	12.2 ± 3.5	−3.382	<0.001^†^	1.05 (1.03–1.08)	<0.001
Neutrophil (10^9^/L)	10.9 ± 3.5	10.5 ± 3.3	−3.046	0.002^†^	1.05 (1.03–1.08)	<0.001
Lymphocyte (10^9^/L)	0.8 ± 0.4	0.8 ± 0.4	1.200	0.231^†^	0.88 (0.91–1.09)	0.232
Platelet (10^9^/L)	137.0 ± 55.0	150.7 ± 55.3	5.800	<0.001^†^	0.99 (0.99–1.00)	<0.001
NLPR	12.4[7.5, 20.0]	10.6[6.4, 16.7]	N/A	<0.001^‡^	1.02 (1.02–1.03)	<0.001

^a^OR was adjusted by age, gender, and body mass index.

^†^Refers to Student’s *t* test (for continuous variables which were normally distributed).

^‡^Refers to Wilcoxon test (for continuous variables which were non-normally distributed).

AKI: acute kidney injury; aOR: adjusted odds ratio; ICU: intensive care unit；NLPR: neutrophil-to-platelet*lymphocyte ratio.

### Dose-response relationship of NLPR and AKI risk

**Figure 1.**
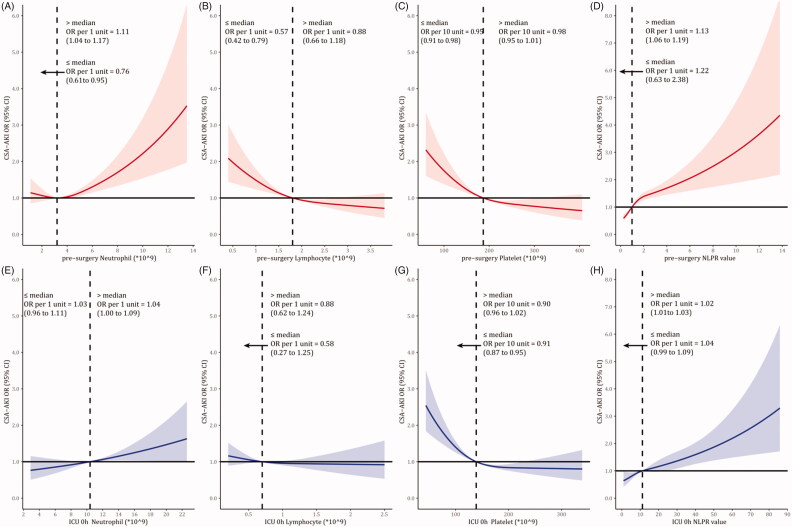
Dose-response relationship of CSA-AKI and peripheral blood cell counts and NLPR.

In Figure 1, we used restricted cubic splines to visualize the dose-response relationship of blood cells with CSA-AKI incidence. Considering the ‘U’ shape relationship between preoperative neutrophil counts and AKI, the plot showed a decrease of the risk within the lower range of neutrophil counts, which reached the lowest risk around the median (3.2*10^9^). It then increased after that (p for non-linearity <0.001). The OR per 1 unit increase was 0.76 (95% CI 0.61–0.95) below 3.2*10^9^, and 1.11 (95% CI 1.04–1.17) above 3.3*10^9^. The risk of CSA-AKI was relatively flat until around the median of lymphocyte, platelet, NLPR. It then started to increase rapidly afterward. The median of preoperative NLPR was 1.0. Above the median, the OR per 1 unit increase was 1.13 (95% CI 1.06–1.19). In comparison, no significant association was found below the median (OR 1.22 95% CI 0.63–2.38). Similarly, we observed such a ‘J’ shape curve between postoperative NLPR and AKI. The OR increased along with the NLPR increase after surgery below the median of 11.0 (OR 1.02 95% CI 1.01–1.03). Then we classified the NLPR value into five groups (Table 3). Compared with the preoperative NLPR level of 0.50–0.99, AKI risk kept growth in higher NLPR groups (the adjusted OR increased from 1.23 to 2.61). As for postoperative NLPR, the incidence was increased remarkably from 31.6% in the reference group to 46.2% in the highest group (aOR rose from 1.13 to 2.09).

**Table 3.** Perioperative NLPR levels and their association with CSA-AKI (*n* = 2449).

	Total	AKI (%)	aOR (95% CI)^a^	*p*-Value
*NLPR (pre-surgery)*				
≤0.49	236	69 (29.2)	1.09 (0.78–1.52)	0.932
0.50–0.99	1005	291 (29.0)	Ref	–
1.00–1.99	843	296 (35.1)	1.23 (1.00–1.51)	0.054
2.00–2.99	200	95 (47.5)	1.80 (1.30–2.49)	<0.001
≥3.00	165	87 (52.7)	2.61 (1.85–3.69)	<0.001
*NLPR (post-surgery)*				
≤4.90	346	97 (28.0)	0.78 (0.58–1.05)	0.107
5.00–9.99	727	230 (31.6)	Ref	–
10.00–19.99	889	301 (33.9)	1.13 (0.91–1.41)	0.278
20.00–29.99	290	119 (41.0)	1.69 (1.26–2.27)	0.001
≥30.00	197	91 (46.2)	2.09 (1.49–2.93)	<0.001

^a^OR was adjusted by age, gender, and body mass index.

AKI: acute kidney injury; aOR: adjusted odds ratio; NLPR: neutrophil to platelet*lymphocyte ratio.

**Table 4.** Antibiotic treatment and the risk of CSA-AKI in high NLPR groups (*n* = 365).

Antibiotic use	Total		NLPR (2.00–2.99)		NLPR (≥3.00)	
	AKI	aOR	AKI	aOR	AKI	aOR
	(%)	(95% CI)^a^	(%)	(95% CI)^a^	(%)	(95% CI)^a^
Yes	27	0.44	10	0.39	17	0.44
	(32.9)	(0.26–0.76)	(29.4)	(0.16–0.95)	(35.4)	(0.21–0.91)
No	155	Ref	85	Ref	70	Ref
	(54.8)		(51.2)		(59.8)	

^a^OR was adjusted by age, gender, and body mass index.

AKI: acute kidney injury; aOR: adjusted odds ratio; NLPR: neutrophil to platelet*lymphocyte ratio.

### Preoperative antibiotic use in patients with high NLPR

We further retrieved the medication data, especially the antibiotic use, in 365 patients whose preoperative NLPR was over 2.0 (Table 4). Of them, 22.4% (*n* = 82) had ever used antibiotics before cardiac surgery. The incidence of AKI in patients receiving antibiotic treatment was 32.9%, significantly lower than that without antibiotics (54.8%, *p* < 0.001). The aOR was estimated as 0.44 (95% CI 0.26–0.76). Subgroup analysis revealed that patients in the second-highest NLPR group (2.00–2.99) could benefit more from antibiotic treatment (aOR = 0.39, 95% CI 0.16–0.95).

### Prediction model of CSA-AKI based on perioperative NLPR

**Figure 2.**
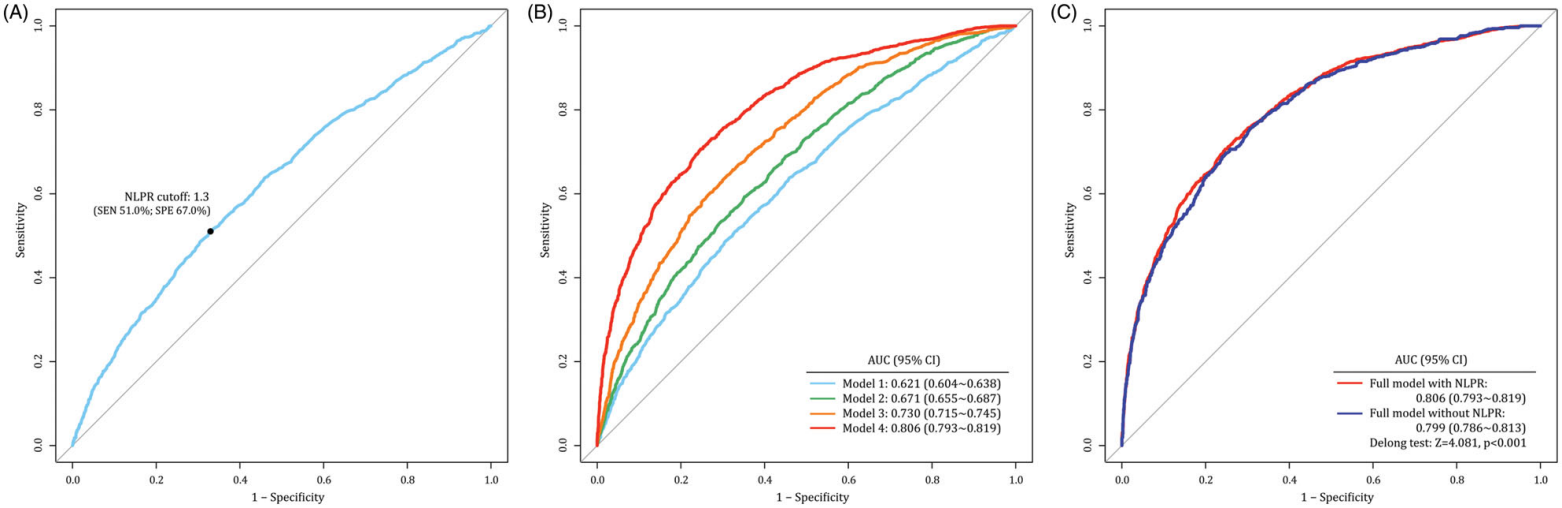
Prediction model of CSA-AKI through the generalized estimating equation (GEE). (3A: the optimal NLPR cutoff was set at 1.3; 3B: model 1 only enrolled the NLPR values in two-time points, model2 was model 1 plus demographic factors; model 3 was model 2 plus preoperative factors, model 4 was model 3 plus intra-/postoperative factor; 3C: comparison between the model with and without NLPR).

We combined the NLPR values in two-time points to predict CSA-AKI occurrence (Figure 2). Considering the correlation among repeated measures, we applied the generalized estimating equation (GEE) for multiple analyses. In the model with NLPR alone, the AUC value was 0.621, 95%CI 0.604–0.638. The optimal cutoff of NLPR was set at 1.3, which has a sensitivity of 51.0% and specificity of 67.0%. With the enrollment of variables progressively, the AUC values increased from 0.671 (95% CI 0.655–0.687) to 0.730 (95% CI 0.715–0.745), and then to 0.806 (95% CI 0.793–0.819). Moreover, we also evaluated the attribution of NLPR to AKI in the full model. Delong test revealed that the AUC of model with NLPR was significantly higher than the AUC without NLPR (AUC: 0.806 vs. 0.799, *Z* = 4.081, *p* < 0.001).

## Discussion

The present study provides the first report for integrating the dynamics of NLPR at two-time points into the prediction model and described the dose-response relationship of NLPR and AKI risk. Previous studies also identified that NLPR was associated with AKI in sepsis [22] and abdominal surgery [23]. Compared with non-AKI patients, we found that perioperative neutrophil counts and NLPR were higher in AKI patients. The relationship between preoperative neutrophils counts and AKI was not linear but showed a ‘U’ shape. Restricted cubic splines showed a decrease of the risk within the lower range of neutrophil counts, which reached the lowest risk around the median and then increased after that. Neutrophil aggregation is reported acting as a subclinical inflammation stage, while neutropenia reflects a physiological condition that the body may be in disorder, such as serious inflammation or myelosuppression [24,25]. The effect of preoperative and postoperative NLPR on AKI was a ‘J’ shape. CSA-AKI’s risk was relatively flat until 1.0 of preoperative NLPR and increased rapidly afterward, with an odds ratio of 1.13 per 1 unit.

Neutrophils and lymphocytes play essential roles in the innate immune system and adaptive immune system, respectively [26]. At the early stage of inflammation, neutrophils are the first cells recruited from blood to tissue. It can phagocytose pathogens and particles, generate reactive oxygen and nitrogen species, and release antimicrobial peptides, which strengthen the innate immune system. In the present study, we found that, per 1 unit neutrophils increase, the OR of CSA-AKI was 1.11 (95% CI 1.04–1.17), indicating that subclinical neutrophilia also played an important role in the pathogenesis of AKI. It might be explained by the high proportion of ‘primed for recruitment’ neutrophils. Noah *et al.* found that a distinct population of primed neutrophils in circulation had a high expression of adhesion and activation markers [27]. Additionally, previous experimental studies revealed that T helper 1 (Th1) and T helper 2 (Th2) cells can contribute to the pathophysiology of AKI [28]. During the process of inflammation, free platelets could bind to neutrophils to form platelet-neutrophil aggregates (PNA) [29]. Substances released by PNA also induce neutrophils tissue invasion, inflammatory mediator release and exacerbate local or systemic inflammatory response.

Cardiovascular surgery involves a series of renal injury pathways. It includes hypoperfusion, ischemia-reperfusion injury, neurohumoral activation, inflammation and oxidative stress [30]. CPB are associated with the activation of multiple inflammatory pathways and the increase pro-inflammatory cytokines. Neutrophil infiltration is detected in post-ischemic mouse kidneys [31,32] and biopsy samples from patients with early AKI participate in the pathogenesis of renal injury following IRI [33,34]. The possible mechanism is that neutrophils participate in the induction of renal injury by obstructing the renal microvasculature and secreting oxygen free radicals and proteases [25]. The adhesion and accumulation of activated platelets within the vascular beds of renal tissue early after IR can act as a bridge between leukocytes and the endothelial wall, thereby enhancing leukocyte recruitment, activation and extravasation [35,36]. Consistent with our study, reduced platelet counts were associated with a higher incidence of AKI [37,38]. The main reason for thrombopenia is the absolute decrease of platelet amount and/or the relative decrease of free platelet proportion. On one hand, the total amount of platelets decreased when patients suffered from malnutrition or receiving a prolonged ACCT during cardiovascular surgery. This ischemia reperfusion injury has been proved as the main pathophysiological mechanism of CSA-AKI. On the other hand, the PNA can lead to vascular lesions and tissue destruction in kidney.

In this study, we creatively incorporated platelets into traditional NLR and the new indicator was used to quantify the effect of inflammation on AKI. After integrating the dynamic NLPRs into multiple predictive models, we found that the AUC was up to 0.806, higher than the AUC without NLPR. It will facilitate AKI risk classification management and allow clinicians to intervene AKI in early-stage. While the model’s specificity was superior to sensitivity. It suggests that NLPR could be most effectively used to exclude AKI. For high-risk patients, further testing (SCr or novel biomarkers) is necessary to stratify their AKI risk. Notably, we found that the incidence of AKI in patients receiving antibiotic treatment was significantly lower than that without antibiotics. This phenomenon may be related to blocking certain inflammatory processes by antibiotics. Vancomycin and ceftriaxone have been proved to be potentially nephrotoxic. However, these antibiotics are still the first-line antibiotic for the infection after heart surgery. Subgroup analysis revealed that the application of nephrotoxic antibiotics could reduce the risk of CSA-AKI. However, cardiac surgeons and nephrologists should reach a consensus and weigh up the pros and cons of nephrotoxic antibiotics.

Still, our study has several limitations to be declared. Firstly, this study was designed as a single-center study. And it would be better to follow-up the patients and describe the relation between NLPR and long-term kidney recovery. Secondly, we did not compare it with C-reactive protein and monitor the related cytokine expression, such as interleukin (IL)-8, myeloperoxidase, and IL-6. However, our pilot study showed that NLPR had a highly positive correlation to procalcitonin and lactic acid. In future studies, a large-scale cohort and multi-omics analysis are needed to verify the predictive value of NLPR in CSA-AKI incidence.

In conclusion, the dynamics of perioperative NPLR is a promising and noninvasive marker relating to acute kidney injury during the perioperative period. Protective strategies should be implemented for those who had initially increased NLPR. It will facilitate AKI risk management and allow clinicians to intervene early so as to reverse renal damage.

## Supplementary Material

Supplemental Material
