# Supplementary figures and images for: Dynamics in perioperative neutrophil-to-lymphocyte*platelet ratio as a predictor of early acute kidney injury following cardiovascular surgery
Source: Ren Fail. 2021 Jun 30;43(1):1012–9. doi: 10.1080/0886022X.2021.1937220 (PMC8260043; doi:10.1080/0886022X.2021.1937220)

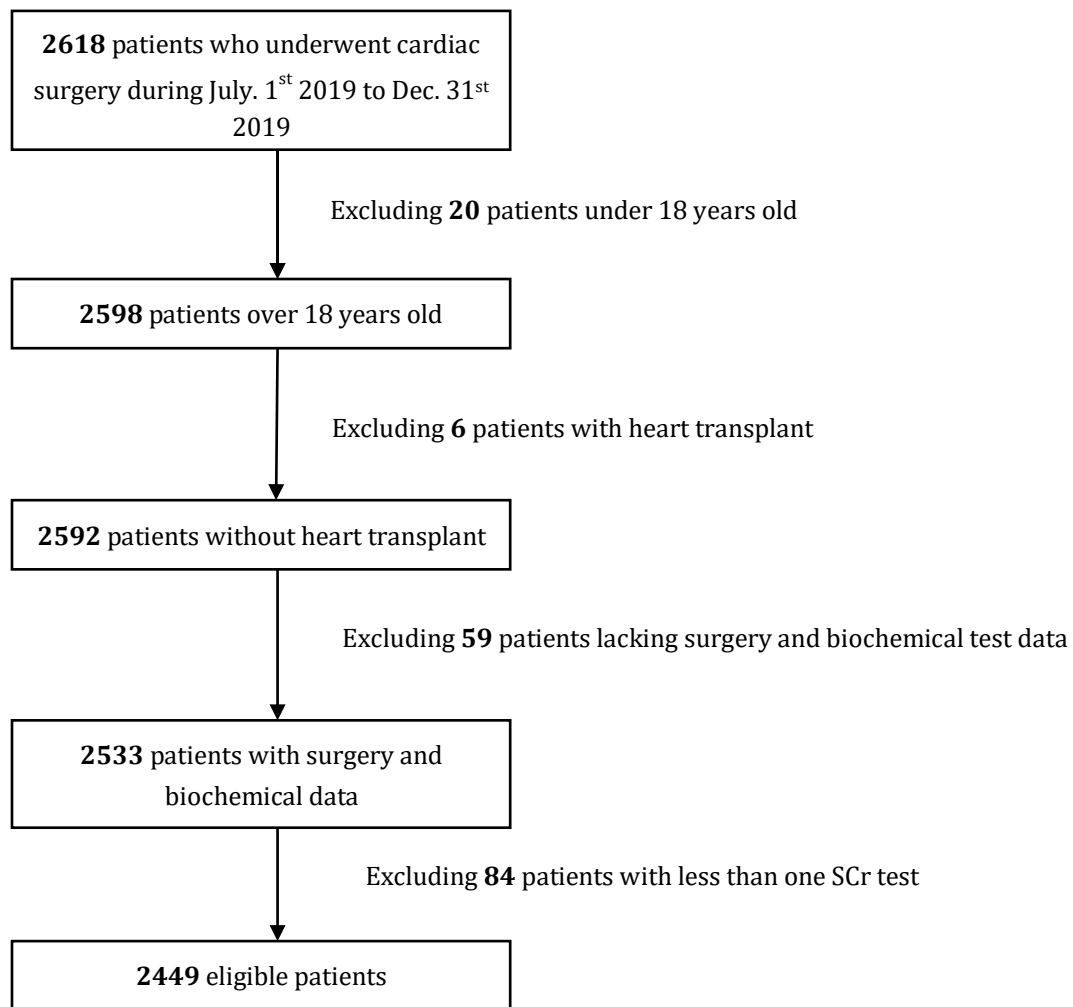

**Supplement Figure 1. Flow chart of the study population selection.**

Supplement: Supplemental Material [file IRNF_A_1937220_SM5227.pdf]
